# Mechanical force-driven TNFα endocytosis governs stem cell homeostasis

**DOI:** 10.1038/s41413-020-00117-x

**Published:** 2021-01-01

**Authors:** Wenjing Yu, Chider Chen, Xiaoxing Kou, Bingdong Sui, Tingting Yu, Dawei Liu, Runci Wang, Jun Wang, Songtao Shi

**Affiliations:** 1 Department of Anatomy and Cell Biology, School of Dental Medicine, University of Pennsylvania, Philadelphia, PA 19104 USA; 2 State Key Laboratory of Oral Diseases, National Clinical Research Center for Oral Diseases, West China Hospital of Stomatology, Sichuan University, Chengdu, 610041 Sichuan China; 3 South China Center of Craniofacial Stem Cell Research, Guanghua School and Hospital of Stomatology, Sun Yat-sen University, Guangzhou, 510055 Guangdong China; 4 Research and Development Center for Tissue Engineering, School of Stomatology, Fourth Military Medical University, Xi’an, 710032 Shaanxi China; 5 Department of Orthodontics, Peking University School and Hospital of Stomatology, 100081 Beijing, China

**Keywords:** Bone quality and biomechanics, Bone

## Abstract

Mesenchymal stem cells (MSCs) closely interact with the immune system, and they are known to secrete inflammatory cytokines in response to stress stimuli. The biological function of MSC-derived inflammatory cytokines remains elusive. Here, we reveal that even under physiological conditions, MSCs produce and release a low level of tumor necrosis factor alpha (TNFα), which is unexpectedly required for preserving the self-renewal and differentiation of MSCs via autocrine/paracrine signaling. Furthermore, TNFα critically maintains MSC function in vivo during bone homeostasis. Mechanistically, we unexpectedly discovered that physiological levels of TNFα safeguard MSC homeostasis in a receptor-independent manner through mechanical force-driven endocytosis and that endocytosed TNFα binds to mammalian target of rapamycin (mTOR) complex 2 and restricts mTOR signaling. Importantly, inhibition of mTOR signaling by rapamycin serves as an effective osteoanabolic therapeutic strategy to protect against TNFα deficiency and mechanical unloading. Collectively, these findings unravel the physiological framework of the dynamic TNFα shuttle-based mTOR equilibrium that governs MSC and bone homeostasis.

## Introduction

Mesenchymal stem cells (MSCs), heterogeneous primitive cells that were originally discovered in the adult bone marrow stroma, possess self-renewal and multidifferentiation potential and are critical for maintaining homeostasis in multiple tissues/organs.^1^ Understanding the regulatory mechanisms of MSC behaviors under physiological and pathological conditions represents a fundamental scientific issue with implications for deciphering disease pathogenesis and establishing appropriate stem cell-based therapeutics.^1^ In particular, the notion that MSCs closely interact with the immune system has increasingly been recognized, and the function of MSCs has been shown to be tightly governed by multiple inflammatory cytokines.^2,3^ We have previously reported that tumor necrosis factor alpha (TNFα) and interferon gamma (IFNγ) synergistically impair MSC stemness in the context of osteopenia and diminished tissue regeneration,^3,4^ whereas others have documented that the immunomodulatory effect of MSCs is mediated by IFNγ combined with TNFα and interleukins (ILs).^5,6^ Interestingly, in addition to the various immunosuppressive factors they produce,^2^ MSCs are also known to express and secrete certain inflammatory cytokines, which have been reported to respond to extrinsic stresses, such as hypoxia and immune mediators.^7–10^ However, whether MSCs are capable of releasing inflammatory cytokines under physiological conditions and what the functional purposes of MSC-derived inflammatory cytokines might be remain unclear.

The production, secretion, and functional execution of inflammatory cytokines involve a series of fine-tuned molecular mechanisms.^11^ These mechanism are particularly elaborate for TNFα, the prototypic member of the TNF superfamily ligands; TNFα plays a crucial role in regulating a variety of biological processes, not only inflammation.^11,12^ Initially expressed after translation in T lymphocytes, macrophages and other types of cells, TNFα exists as a 26 kD membrane-anchored precursor (pro-TNFα), the extracellular domain of which is cleaved by metalloproteinases, specifically the TNFα-converting enzyme (TACE), to yield the 17 kD mature TNFα.^13^ Biologically active TNFα transduces signals in an autocrine/paracrine manner through two receptors, TNFR1 and TNFR2, and activates downstream cascades, including nuclear factor kappaB (NFκB) transcriptional signaling.^14^ Intriguingly, the effects of TNFα are dependent on the recipient cell types and the dosage and duration of TNFα application. For MSCs, it has been reported that high concentrations of TNFα (over 1 ng·mL^−1^) in culture suppress osteogenic potential via NFκB activation, while lower doses of TNFα show benefits to some extent.^4,15^ Furthermore, in aging- and disease-associated microenvironments in vivo upregulated TNFα levels are detected in the circulation and in the bone marrow space, resulting in resident MSC impairment and osteopenic phenotypes.^4,16^ Therefore, it is safe to assume that TNFα needs to reach pathological levels to exert detrimental effects on MSCs. Whether and how physiological levels of TNFα might regulate MSCs via potentially distinct mechanisms is an interesting and significant question that warrants investigation.

Mechanical stimuli, including physical cues from the matrix and applied forces, represent another critical extrinsic factor that controls MSC fate, particularly in bone, where the response to mechanical loading is constantly adapted and coordinated.^17^ In this regard, bone marrow MSCs (BMMSCs) are especially mechanosensitive and mechanoresponsive to support their functional regulation and bone maintenance.^18^ Importantly, the mechanical unloading experienced by astronauts in spaceflight and bedridden patients leads to progressive bone loss, but therapeutics have yet to be established.^19^ Accordingly, hindlimb suspension in rodents recapitulates the osteopenic phenotype seen in human unloading, in which BMMSCs exhibit osteogenic inhibition due to coordinated responses of multiple signaling pathways.^20^ Notably, mechanical unloading also induces general humoral alterations, including the onset of systemic inflammation, with increased susceptibility to autoimmune disorders.^21^ Further dissecting the mechanisms underlying the mechanical regulation of MSCs via potential inflammatory cytokine reactions would be helpful for identifying novel molecular targets for counteracting unloading-induced osteopenia.

In this study, we aimed to investigate the physiology and mechanisms of the interaction between MSCs and inflammatory cytokines and to examine the potential pathophysiological and therapeutic contributions of this process to mechanical regulation. Surprisingly, we reveal that MSCs are capable of producing and releasing a relatively low level of TNFα under physiological conditions and that physiological levels of TNFα are indispensable for the functional maintenance of MSCs both in vitro and in vivo. Mechanistically, we further discovered a receptor-independent mechanism by which TNFα exerts its effects through endocytosis, which is driven by mechanical force and safeguards MSC homeostasis based on the uptake of TNFα, restricting the activation of mammalian target of rapamycin (mTOR) signaling. Importantly, inhibition of mTOR signaling by rapamycin serves as an effective osteoanabolic therapeutic strategy to protect against TNFα deficiency and mechanical unloading. Collectively, these findings unravel the physiological framework by which the dynamic TNFα shuttle-based mTOR equilibrium governs MSC and bone homeostasis.

## Results

### MSCs produce and release TNFα for functional maintenance

**Fig. 1:**
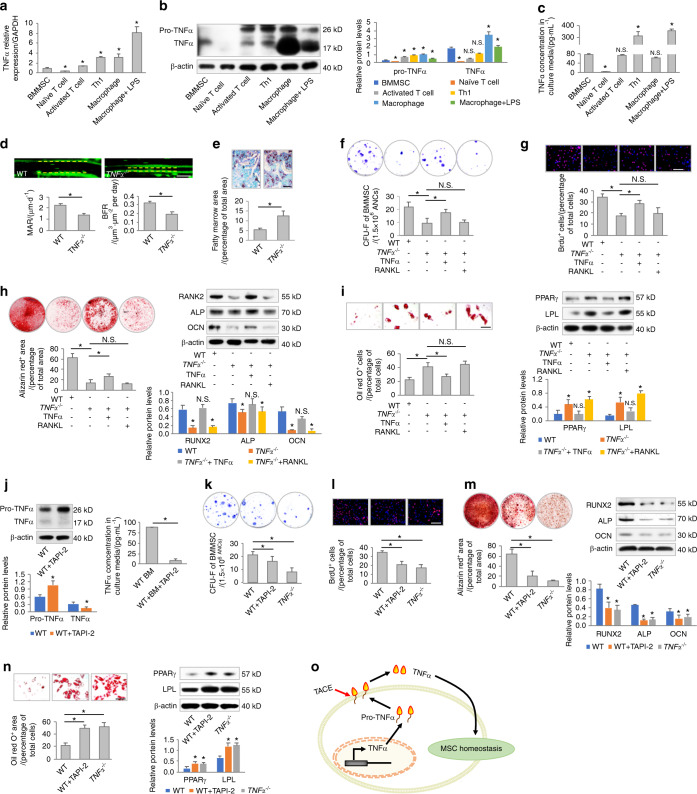
MSCs produce and release TNFα for functional maintenance. **a**–**c** Examination of mRNA expression levels, protein expression levels, and secreted concentrations of TNFα in different cell types. *N* = 3. For quantification of Western blotting, two-tailed Student’s *t* test was used for comparisons between different cell types and MSCs. **d** Calcein labeling for bone formation analysis in WT and *TNFα*^*−/−*^ mice (*N* = 5). Scale bar = 50 μm. **e** Oli red O staining for bone marrow adiposity in WT and *TNFα*^*−/−*^ mice (*N* = 5). Scale bar = 150 μm. **f**–**i** Functional analyses of MSCs according to CFU, BrdU labeling, osteogenic, and adipogenic differentiation. TNFα and RANKL were added at 1 ng·mL^−1^. *N* = 5. Scale bars = 100 μm. **j** Examination of protein expression levels and secreted concentrations of TNFα. *N* = 3. TAPI-2 was used to inhibit TACE at 120 nmol·L^−1^. **k**–**n** Functional analyses of MSCs according to CFU, BrdU labeling, and osteogenic and adipogenic differentiation. *N* = 3. Scale bars = 100 μm. **o** Diagram showing the production and release of TNFα for the functional maintenance of MSCs. For quantification of Western blotting, a two-tailed Student’s *t* test was used for the comparison between the treatment and WT groups. *P < 0.05. N.S. not significant. Data represent the mean ± SD

To investigate whether and to what extent MSCs are capable of producing inflammatory cytokines under physiological conditions, we cultured mouse BMMSCs and analyzed their production of TNFα, which is one of the most widely studied inflammatory cytokines with a variety of biological functions.^11^ A combination of quantitative real-time polymerase chain reaction (qRT-PCR), Western blot analysis, and enzyme-linked immunosorbent assay (ELISA) was applied to detect the mRNA expression, protein expression, and secretion of TNFα (Fig. 1a–c). TNFα production by MSCs was evaluated in comparison with the levels produced by naïve and activated T cells, T helper 1 (Th1) cells, and macrophages with and without lipopolysaccharide (LPS) treatment (Fig. 1a–c). The data demonstrated that MSCs indeed expressed both pro-TNFα and TNFα, and they released a certain amount of TNFα into the culture medium, which was more than the amount of TNFα released by naïve T cells but much less than the amount of TNFα released by Th1 cells and LPS-activated macrophages during the same period (Fig. 1a–c).

To address whether physiological TNFα is required for the functional maintenance of MSCs, we first examined bone formation and bone marrow adiposity, which indicate the osteogenic and adipogenic behaviors of tissue-resident MSCs,^22,23^ in wild-type (WT) and *TNFα*^*−/−*^ mice. Calcein labeling analysis showed that *TNFα*^−^^*/*−^ mice suffered from a decrease in bone formation, as demonstrated by a lower mineral apposition rate (MAR) and bone formation rate (BFR) compared to those of WT mice (Fig. 1d). On the other hand, *TNFα*^*−/−*^ mice exhibited higher percentages of fatty marrow area than WT mice (Fig. 1e). To directly evaluate whether MSC function was dysregulated under TNFα deficiency, we isolated BMMSCs from WT and *TNFα*^*−/−*^ mice (Fig. S1A, B) and analyzed their behaviors ex vivo in terms of colony formation, proliferation, and osteogenic and adipogenic differentiation (Fig. 1f–i).^22,24^ The data confirmed that *TNFα*^*−/−*^ MSCs were less capable of forming fibroblastic colonies (Fig. 1f), incorporating bromodeoxyuridine (BrdU) (Fig. 1g) during proliferation and differentiating into mineralizing osteoblasts than were WT MSCs (Fig. 1h), while they were misdirected toward adipocytes during differentiation (Fig. 1i). Interestingly, MSCs derived from mice lacking *IFNγ* and *IL-6*, two additional potentially important inflammatory regulators of MSC function,^10,25^ did not show functional impairments (Fig. S1C–G), suggesting that TNFα-dependent MSC regulation occurs without general effects of inflammatory cytokines.

Next, we intended to determine whether TNFα per se maintains MSC function by treating cultured *TNFα*^*−/−*^ MSCs with TNFα. Dose-effect analyses demonstrated that a low dose of 1 ng·mL^−1^ TNFα mimicked the physiological concentration of TNFα secreted by WT MSCs (Fig. S2A). Furthermore, 1 ng·mL^−1^ and lower doses of TNFα exhibited a limited ability to activate TNFR downstream signaling pathways, which were previously revealed to be detrimental for MSCs^3,4^ (Fig. S2B–D). Therefore, we applied TNFα at a concentration of 1 ng·mL^−1^ and explored whether this low “physiological” level of TNFα would be beneficial for MSCs. We further used receptor activator of nuclear factor kappaB ligand (RANKL), another member of the TNF superfamily of cytokines that is important for bone homeostasis,^26^ at the same dose of 1 ng·mL^−1^ to treat MSCs as a control for TNFα. We discovered that 1 ng·mL^−1^ TNFα, but not RANKL, was effective in rescuing the functional decline of MSCs derived from *TNFα*^*−/−*^ mice, as proven by increases in colony formation, proliferation, and osteogenic differentiation with inhibition of adipogenesis (Fig. 1f–i). Additional in vitro experiments using genetic overexpression of *TNFα* in *TNFα*^*−/−*^ MSCs confirmed that TNFα is functionally important for preserving homeostasis of MSCs (Fig. S3A–D). Further knockdown of *TNFα* using a small interfering RNA (siRNA) followed by extrinsic TNFα rescue also revealed that TNFα is indispensable for MSC homeostasis (Fig. S3E–H).

The initially expressed transmembrane pro-TNFα can be specifically cleaved by TACE to release soluble, biologically active, mature TNFα.^13^ Intriguingly, pro-TNFα failed to rescue the deficient function of MSCs derived from *TNFα*^−^^*/−*^ mice, suggesting that TNFα regulation of MSCs was based on the mature form (Fig. S4A–D). Therefore, we further investigated whether the release of mature TNFα is required for the functional regulation of MSCs by using TNF protease inhibitor-2 (TAPI-2), a hydroxamate-based inhibitor of TACE.^27^ We confirmed that TAPI-2 application elevated the intracellular protein level of pro-TNFα while remarkably suppressing MSC release of TNFα into the culture medium (Fig. 1j). We further revealed that TAPI-2 treatment in WT MSCs induced functional decline, mimicking the phenotype of *TNFα*^*−/−*^ MSCs (Fig. 1k–n). Taken together, the above results indicated that MSCs produced and released TNFα for their functional maintenance (Fig. 1o).

### TNFα safeguards MSC homeostasis in a receptor-independent manner through endocytosis

**Fig. 2:**
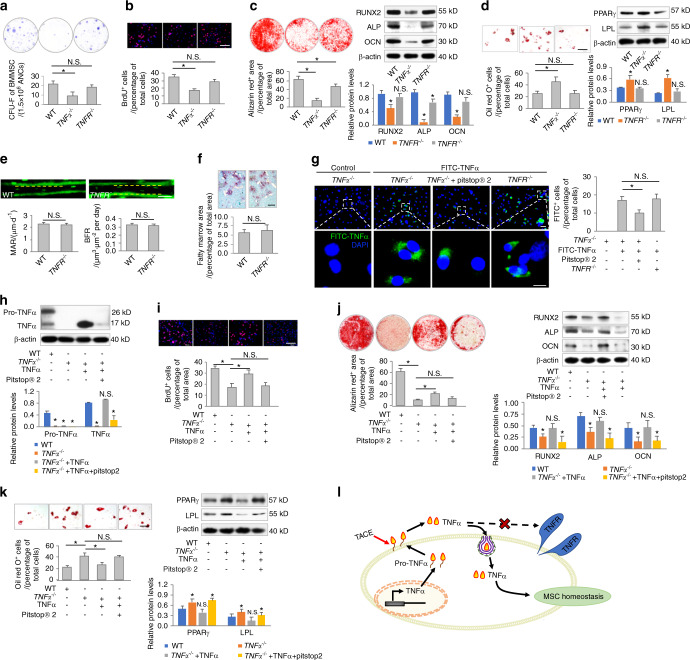
TNFα safeguards MSC homeostasis in a receptor-independent manner through endocytosis. **a**–**d** Functional analyses of MSCs according to CFU, BrdU labeling, and osteogenic and adipogenic differentiation. MSCs were derived from WT, *TNFα*^*−/−*^ or *TNFR*^*−/−*^ mice (*N* = 5). Scale bars = 100 μm. **e** Calcein labeling for bone formation analysis in WT and *TNFR*^*−/−*^ mice (*N* = 5). Scale bar = 50 μm. **f** Oli red O staining for bone marrow adiposity in WT and *TNFR*^*−/−*^ mice (*N* = 5). Scale bar = 150 μm. **g** Endocytosis analysis of FITC-labeled TNFα uptake by MSCs for 24 h in vitro. Pitstop^®^ 2 was used to inhibit clathrin-mediated endocytosis at 12 μmol·L^−1^. *N* = 3. Scale bars = 20 μm (top) and 7 μm (bottom). **h** Western blot analysis of protein expression levels (*N* = 3). **i**–**k** Functional analyses of MSCs according to BrdU labeling and osteogenic and adipogenic differentiation. *N* = 3. Scale bars = 100 μm. **l** Diagram showing that TNFα regulates MSC homeostasis in a receptor-independent manner through endocytosis. For quantification of Western blotting, a two-tailed Student’s *t* test was used for the comparison between the treatment and WT groups. *P < 0.05. N.S. not significant. Data represent the mean ± SD

Next, we explored how TNFα regulates MSC homeostasis. Exogenously added TNFα at a concentration of 1 ng·mL^−1^ was able to establish a physiological TNFα microenvironment to maintain MSC function without activating canonical TNFR downstream signaling in cultured MSCs (Fig. S2A, B). To further evaluate whether TNFR contributes to the regulation of MSCs, we isolated MSCs from *TNFR*^*−/−*^ mice and analyzed their function compared to that of WT and *TNFα*^*−/−*^ MSCs (Figs. 2a–d and S1A, B). The data demonstrated that the colony-forming and proliferative capacities of *TNFR*^*−/−*^ MSCs were not significantly decreased compared to those of WT MSCs (Fig. 2a, b). Moreover, although *TNFR*^−^^*/−*^ MSCs were less capable of osteogenesis than WT MSCs, *TNFα*^*−/−*^ MSCs were deficient in osteogenic differentiation (Fig. 2c). In addition, *TNFR*^*−/−*^ MSCs showed similar adipogenic differentiation to WT MSCs (Fig. 2d). These results indicated limited effects of TNFR on MSC function, which were further confirmed by in vivo analyses of bone formation and bone marrow adiposity, revealing that WT and *TNFR*^*−/−*^ mice were comparable (Fig. 2e, f).

In deciphering how TNFα regulates MSC homeostasis in a receptor-independent manner, we noticed that when *TNFα*^*−/−*^ MSCs were treated with TNFα, the levels of TNFα detected in the culture medium were always much lower than what we originally added (Fig. S2A) and that intracellular TNFα could be found (Fig. S2B). These data indicated that TNFα might be taken up by deficient MSCs despite the lack of receptor activation. To address this hypothesis, we labeled TNFα with fluorescein isothiocyanate (FITC) for MSC treatments and discovered that FITC-TNFα was indeed taken up by *TNFα*^−^^*/−*^ MSCs (Fig. 2g). To examine whether TNFα uptake was mediated by endocytotic processes, we used Pitstop^®^ 2, an inhibitor of clathrin-mediated endocytosis.^28^ We identified that Pitstop^®^ 2 effectively inhibited, but did not block, TNFα uptake by *TNFα*^*−/−*^ MSCs, suggesting that TNFα uptake occurred at least partially through clathrin-mediated endocytosis (Fig. 2g). We further found that TNFα uptake was not affected by TNFR deficiency, indicating a receptor-independent mechanism (Fig. 2g).

Next, we examined whether TNFα endocytosis regulates MSC function. Western blot analysis confirmed that Pitstop^®^ 2 treatment efficiently suppressed the uptake of TNFα into *TNFα*^*−/−*^ MSCs (Fig. 2h). As expected, Pitstop^®^ 2 treatment further significantly diminished the ability of TNFα to rescue *TNFα*^*−/−*^ MSCs, leading to dysregulation of proliferation and differentiation (Fig. 2i–k). The above results collectively suggested that TNFα safeguarded MSC homeostasis in a receptor-independent manner through endocytosis (Fig. 2l).

### Endocytosed TNFα binds to mTORC2 and restricts the activation of mTOR signaling

**Fig. 3:**
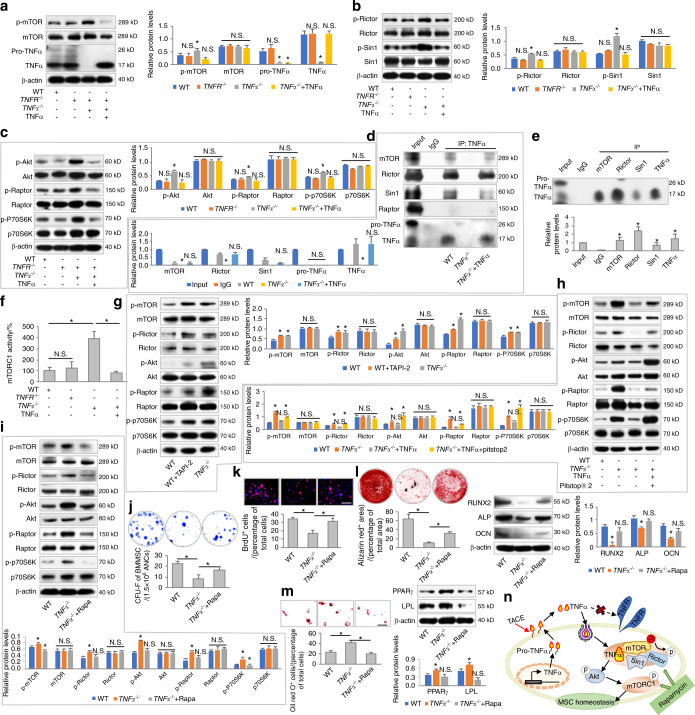
Endocytosed TNFα binds to mTORC2 and restricts activation of mTOR signaling for functional regulation of MSCs. **a**–**c** Western blot analyses of mTOR signaling in response to *TNFR* or *TNFα* deficiency. *N* = 3. TNFα was added at 1 ng·mL^−1^. **d**, **e** Co-IP analyses of the binding of TNFα to mTOR complex components (*N* = 3). **f** Analysis of mTORC1 activity using an ELISA-based assay (*N* = 3). **g**–**i** Western blot analyses of mTOR signaling. *N* = 3. TAPI-2, TNFα, Pitstop^®^ 2, and rapamycin (Rapa) were added at 120 nmol·L^−1^, 1 ng·mL^−1^, 12 μmol·L^−1^, and 50 nmol·L^−1^, respectively, in vitro. **j**–**m** Functional analyses of MSCs according to CFU, BrdU labeling, and osteogenic and adipogenic differentiation (*N* = 3). Scale bars = 100 μm. **n** Diagram showing TNFα binding to mTORC2 and restricting activation of mTOR signaling for functional regulation of MSCs. For quantification of Western blotting, a two-tailed Student’s *t* test was used for the comparison between the treatment and WT groups. *P < 0.05. N.S. not significant. Data represent the mean ± SD

Next, we investigated how endocytosed TNFα regulates MSC homeostasis. In screening the multiple signaling pathways that modulate MSC behaviors, we discovered that mTOR signaling changed in response to TNFα deficiency and replenishment (Fig. 3a), whereas the canonical Wnt pathway, the transforming growth factor-beta (TGF-β) pathway and the extracellular signal-regulated kinase (ERK) pathway were not influenced by TNFα (Fig. S5A). In detail, phosphorylation of mTOR (p-mTOR), which indicates pathway activation,^29^ was restricted by TNFα but was not affected by TNFR deficiency in MSCs (Fig. 3a). mTOR functions downstream of phosphoinositide 3-kinase (PI3K) and phosphatase and tensin homolog (PTEN) in two distinct kinase complexes, termed mTOR complex 1 (mTORC1) and complex 2 (mTORC2), and Akt is a downstream target of mTORC2 that regulates mTORC1 activity.^29^ We therefore analyzed the different components of mTOR signaling and found that TNFα inhibited phosphorylation-mediated activation of key mTORC2 subunits, Rictor and Sin1 (Figs. 3b and S3I), without affecting PI3K and PTEN expression in MSCs (Fig. S5B). TNFα also suppressed the phosphorylation of Akt and the key mTORC1 subunit Raptor, as well as the downstream ribosomal protein S6 kinase (P70S6K), in MSCs^29^ (Figs. 3c and S3J).

To decipher how TNFα regulates mTOR signaling in MSCs, we hypothesized that TNFα interacted with mTOR complexes given that the upstream factors PI3K and PTEN were unaffected (Fig. S5B). To address this hypothesis, we performed a protein coimmunoprecipitation (co-IP) assay to examine the potential binding of TNFα with mTOR complexes. We discovered that when using an anti-TNFα antibody for co-IP, mTOR, Rictor and Sin1 could be captured, but Raptor failed to be detected, suggesting that TNFα binds with mTORC2 but not mTORC1 in MSCs (Fig. 3d). Further co-IP analyses using mTOR, Rictor and Sin1 antibodies also captured TNFα in MSCs (Fig. 3e). Interestingly, although TNFα did not directly interact with mTORC1, mTORC1 kinase activity was repressed by TNFα in MSCs, possibly through the restricted activation of Akt (Fig. 3f). We also confirmed that the release and uptake cycle of TNFα contributed to mTOR suppression in MSCs, as inhibiting TACE with TAPI-2 and inhibiting clathrin-mediated endocytosis with Pitstop^®^ 2 resulted in mTOR signaling activation, mimicking the effects of TNFα deficiency (Fig. 3g, h). Additionally, although pro-TNFα could be taken up by MSCs, mTOR activation was not repressed by pro-TNFα, collectively proving that only the mature form of TNFα was capable of regulating mTOR signaling in MSCs (Fig. S4E–H). These data indicated that endocytosed TNFα bound to mTORC2 and restricted the activation of mTOR signaling in MSCs, which might be responsible for the observed functional regulation.

### Rapamycin protects MSC function against TNFα deficiency

The above results prompted us to investigate whether inhibition of mTOR signaling indeed contributes to the functional regulation of MSCs by TNFα and whether pharmaceutical mTOR suppression serves as an effective approach to rescue the functional decline of MSCs under TNFα deficiency. In this regard, we tested rapamycin (Rapa), a potent and specific chemical inhibitor of mTOR complexes and an approved drug in the clinic,^30^ on *TNFα*^*−/−*^ MSCs. We confirmed that rapamycin application suppressed activated mTOR signaling in *TNFα*^*−/−*^ MSCs in vitro (Fig. 3i). Functional analyses of MSCs further identified that rapamycin treatment significantly improved the colony-forming capability, proliferation, and osteogenic differentiation of *TNFα*^*−/−*^ MSCs, while rapamycin inhibited the adipogenic differentiation of *TNFα*^*−/−*^ MSCs, suggesting restoration of the homeostatic condition of MSCs in the context of TNFα deficiency (Fig. 3j–m). These data indicated that mTOR signaling was a critical downstream target of TNFα in regulating MSC homeostasis and a potential therapeutic target for counteracting MSC disorders in the absence of TNFα (Fig. 3n).

**Fig. 4:**
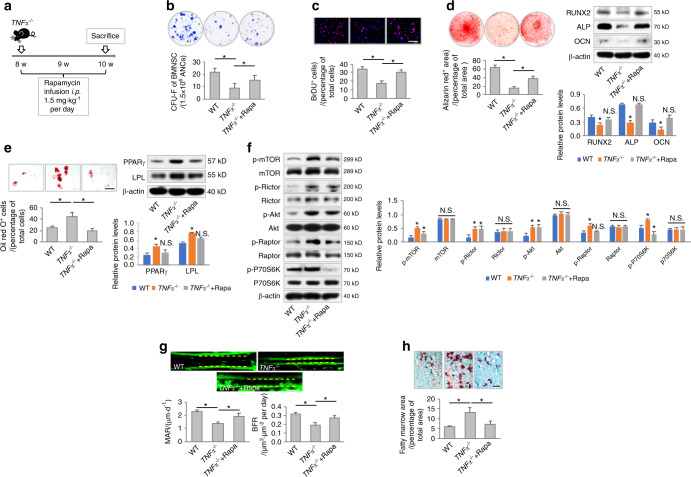
Rapamycin infusion improves MSC function in TNFα^−^^/−^ mice in vivo. **a** Schematic diagram showing the experimental design to investigate the effect of rapamycin treatment on *TNFα*^*−/−*^ mice in vivo. **b**–**e** Functional analyses of MSCs according to CFU, BrdU labeling, and osteogenic and adipogenic differentiation (*N* = 5). Scale bars = 100 μm. **f** Western blot analyses of mTOR signaling (*N* = 3). **g** Calcein labeling for bone formation analysis. *N* = 5. Scale bar = 50 μm. **h** Oli red O staining for bone marrow adiposity. *N* = 5. Scale bar = 150 μm. For quantification of Western blotting, a two-tailed Student’s *t* test was used for the comparison between the treatment and WT groups. *P < 0.05. Data represent the mean ± SD

To further evaluate whether inhibition of mTOR signaling rescues the functional decline of MSCs in *TNFα*^*−/−*^ mice in vivo, we infused rapamycin into *TNFα*^*−/−*^ mice intraperitoneally at a dosage of 1.5 mg·kg^−1^ per day (Fig. 4a); this approach was based on our previous study using rapamycin to ameliorate osteopenia.^30^ After 2 weeks of rapamycin administration, MSCs demonstrated remarkable recovery despite TNFα deficiency, as indicated by the formation of more fibroblastic colonies ex vivo (Fig. 4b). Furthermore, MSCs derived from rapamycin-treated *TNFα*^*−/−*^ mice showed higher proliferative capacity than *TNFα*^*−/−*^ MSCs (Fig. 4c). Regarding differentiation, MSCs derived from rapamycin-treated *TNFα*^*−/−*^ mice showed a preference for osteogenesis rather than adipogenesis (Fig. 4d, e). Molecular examinations of ex vivo MSCs confirmed that rapamycin treatment repressed mTOR signaling under TNFα deficiency (Fig. 4f). Functional recovery of MSCs further contributed to improved bone formation with decreased bone marrow adiposity in rapamycin-treated *TNFα*^*−/−*^ mice (Fig. 4g, h). These results collectively suggested that rapamycin was an effective osteoanabolic therapeutic that protected MSC function against TNFα deficiency.

### Mechanical force drives TNFα endocytosis and promotes MSC function

**Fig. 5:**
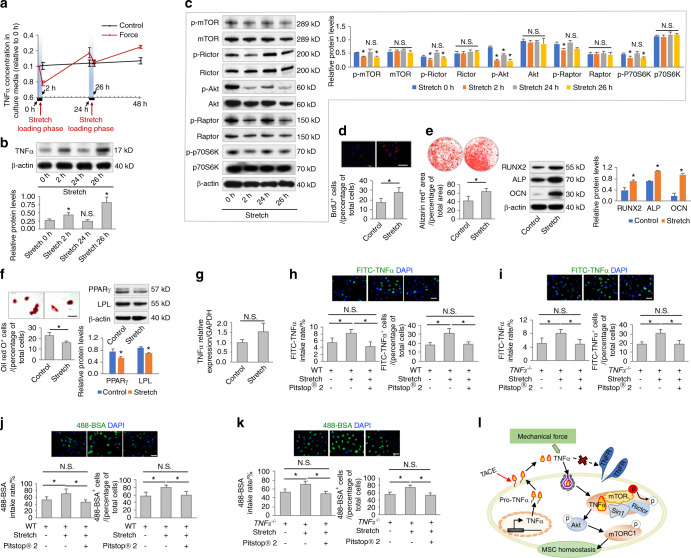
Mechanical force drives TNFα endocytosis and promotes MSC function. **a**, **b** Examination of secreted concentrations and protein expression levels of TNFα. MSCs underwent cyclic loading of stretch force at 15% elongation, 0.5 Hz. *N* = 3. **c** Western blot analyses of mTOR signaling (*N* = 3). **d**–**f** Functional analyses of MSCs according to BrdU labeling and osteogenic and adipogenic differentiation. *N* = 3. Scale bars = 100 μm. Stretch force was applied twice per week for 2 h per session. **g** qRT-PCR analysis of the mRNA expression levels of TNFα (*N* = 3). **h**, **i** Endocytosis analysis of FITC-labeled TNFα uptake by MSCs for 2 h in vitro. Pitstop^®^ 2 was used to inhibit clathrin-mediated endocytosis at 12 μmol·L^−1^. Stretch force was applied for a 2 h period. *N* = 3. Scale bars = 20 μm. **j**, **k** Endocytosis analysis of Alexa Fluor^TM^ 488-conjugated bovine serum albumin (488-BSA) taken up by MSCs for 2 h in vitro. Pitstop^®^ 2 was used to inhibit clathrin-mediated endocytosis at 12 μmol·L^−1^. Stretch force was applied for a 2 h period. *N* = 3. Scale bars = 20 μm. **l** Diagram showing mechanical force-driven TNFα endocytosis. For quantification of Western blotting, a two-tailed Student’s *t* test was used for the comparison between the treatment and stretch 0 h/control groups. *P < 0.05. N.S. not significant. Data represent the mean ± SD

Next, we aimed to investigate how the release and uptake of TNFα are coupled for MSC regulation. MSCs are exposed to a variety of mechanical stimuli in vivo,^18^ which have been reported to influence cellular endocytotic processes.^31,32^ To investigate whether mechanical force regulates TNFα shuttling across the MSC membrane, we applied an intermittent stretch-loading model to cultured MSCs (Fig. 5a). Intriguingly, we discovered that the TNFα concentration in the culture medium of MSCs dropped dramatically during each of the 2 h stretching periods, whereas it gradually increased during mechanical unloading and generally increased with advancing time (Fig. 5a). Correspondingly, intracellular protein levels of TNFα were upregulated after stretching but diminished upon unloading (Fig. 5b). These data suggested constitutive production and release of TNFα by MSCs with inducible internalization triggered by mechanical force.

To determine whether the force-driven dynamics of TNFα contribute to MSC regulation, we examined and revealed changes in mTOR signaling activation in accordance with stretch-provoked TNFα fluctuations (Fig. 5c). Further cellular functional analyses demonstrated that intermittent stretching for 2 h per session twice per week significantly promoted the proliferation and osteogenic differentiation of MSCs, with inhibition of adipogenesis (Fig. 5d–f). As expected, stretch-loading did not induce changes in TNFα mRNA expression (Fig. 5g). Instead, mechanical stretching promoted TNFα uptake in both WT and *TNFα*^*−/−*^ MSCs (Fig. 5h, i). We used Alexa Fluor^TM^ 488-conjugated bovine serum albumin (488-BSA) to confirm that the general endocytosis rate was also boosted by mechanical stretching (Fig. 5j, k). Importantly, when Pitstop^®^ 2 was used to inhibit clathrin-mediated endocytosis, stretch-induced TNFα and 488-BSA internalization was blocked (Fig. 5h–k). To further confirm that mechanical stretching restricted mTOR signaling via TNFα endocytosis, we applied mechanical stretching to *TNFα*^*−/−*^ MSCs and then examined mTOR signaling by Western blot analysis. The results showed that there was no significant alteration in mTOR signaling between the stretch-loading and control groups (Fig. S6A). TNFα treatment followed by mechanical stretching restricted mTOR signaling, whereas this effect was diminished in the presence of Pitstop^®^ 2 (Fig. S6B). Collectively, these results suggested that mechanical force drove TNFα endocytosis and promoted MSC function (Fig. 5l).

### Mechanical unloading induces MSC functional decline and bone loss that are counteracted by rapamycin

**Fig. 6:**
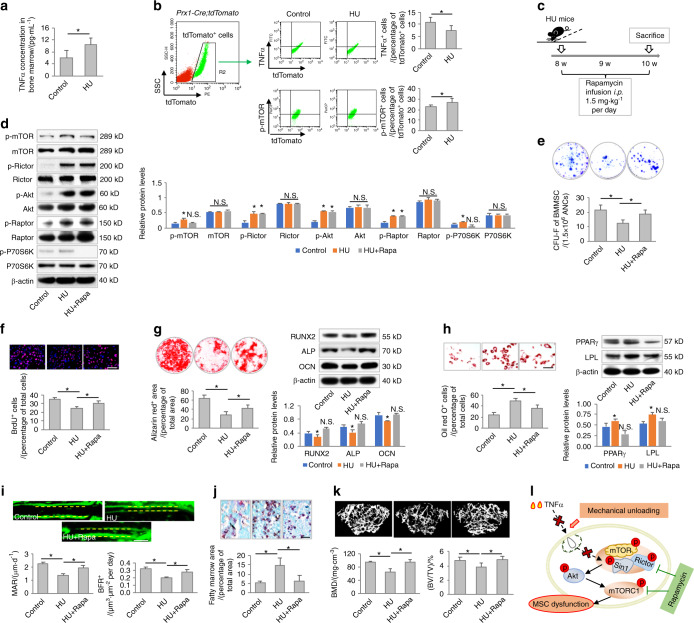
Mechanical unloading induces MSC functional decline and bone loss that are counteracted by rapamycin. **a** ELISA analysis of TNFα levels in the bone marrow of control and hindlimb unloading (HU) mice (*N* = 5). **b** Flow cytometric analyses of TNFα^+^ cells and p-mTOR^+^ cells in bone marrow mesenchymal cells. *Prx1-cre:tdTomato* mice (*N* = 5) were used to label bone marrow mesenchymal cells in vivo. **c** Schematic diagram showing the experimental design used to investigate the effect of rapamycin treatment on HU mice in vivo. **d** Western blot analyses of mTOR signaling (*N* = 3). **e**–**h** Functional analyses of MSCs according to CFU, BrdU labeling, and osteogenic and adipogenic differentiation. *N* = 5. Scale bars = 100 μm. **i** Calcein labeling for bone formation analysis. *N* = 5. Scale bar = 50 μm. **j** Oli red O staining for bone marrow adiposity. *N* = 5. Scale bar = 150 μm. **k** Micro-CT analysis of trabecular bone mass (*N* = 5). **l** Diagram showing mechanical unloading-induced MSC functional decline and bone loss counteracted by rapamycin. For quantification of Western blotting, a two-tailed Student’s *t* test was used for the comparison between the treatment and control groups. *P < 0.05. Data represent the mean ± SD

Inspired by the above data, we intended to investigate the pathophysiological implications of mechanical force-induced TNFα endocytosis. Hindlimb unloading (HU) of mice has been widely used as a ground-based model to simulate the effects of spaceflight and microgravity environments, in which osteopenia and MSC dysfunction have been reported.^20^ Interestingly, we discovered that HU mice were characterized by an elevated level of TNFα in the bone marrow (Fig. 6a). To determine whether TNFα uptake by MSCs was impaired after hindlimb suspension, we applied *Prx1-Cre;tdTomato* mice, in which mesenchymal cells are labeled specifically in vivo. We found that among the bone marrow *tdTomato*^+^ cells, TNFα^+^ cells were indeed less abundant in HU mice than in the control mice, but there was a higher prevalence of p-mTOR^+^ cells, indicating diminished TNFα dynamics with mTOR signaling activation under mechanical unloading (Fig. 6b).

Considering the above findings, we continued to evaluate whether inhibition of mTOR signaling by rapamycin infusion counteracts the putative MSC functional decline in HU mice (Fig. 6c). As expected, rapamycin administration prevented the activation of mTOR signaling in MSCs after hindlimb suspension (Fig. 6d). Furthermore, rapamycin treatment in vivo protected MSCs against HU-induced functional impairments, as indicated by improved colony formation, proliferation, and osteogenic differentiation in MSCs derived from rapamycin-treated mice with suppressed adipogenesis (Fig. 6e–h). Importantly, we revealed that rapamycin infusion substantially prevented the decline of bone formation in HU mice, and rapamycin also rescued bone marrow adiposity (Fig. 6i, j). Finally, we identified rapamycin as an effective therapy for preserving bone mass despite mechanical unloading (Fig. 6k). Collectively, these data indicated that mechanical unloading induced MSC functional decline and bone loss based on TNFα and mTOR signaling dysregulation, and these effects were counteracted by rapamycin (Fig. 6l).

## Discussion

MSCs interact with the immune system for functional modulation and maintenance of tissue/organ homeostasis,^1,5^ while they are known to secrete inflammatory cytokines with unclear implications under physiological conditions.^7–9^ Inflammatory cytokines play crucial roles in regulating various basic biological processes in organisms, whereas the effects and mechanisms of inflammatory cytokines in the context of the stem cell pool remain largely unexplored.^3–5^ In the present study, we revealed that physiological levels of TNFα are needed for functional homeostasis of MSCs via autocrine/paracrine signaling, which is of particular significance for bone maintenance in vivo. We further uncovered a new mechanism mediating the beneficial effects of low levels of TNFα on MSCs, which was proven to be receptor-independent and mediated by mechanical force-driven endocytosis to maintain mTOR signaling equilibrium. Accordingly, to counteract the MSC dysfunction and osteopenia induced by mechanical unloading, rapamycin inhibition of mTOR signaling functions as a downstream therapeutic to protect against TNFα deficiency. Taken together, these findings suggest that dynamic TNFα release and endocytosis safeguard MSC homeostasis and identify an osteoanabolic approach for mechanical unloading.

The regulatory framework of MSC function involves dynamic interactions of MSCs with the surrounding niche, in which MSCs secrete a broad spectrum of cytokines to modulate the niche and respond to various environmental cues for behavioral adaptation.^1,16^ Among the niche factors, immune components, particularly inflammatory cytokines, strongly regulate MSC behaviors in resident and transplanted tissues, and they diminish MSC function in pathophysiological processes to induce tissue deterioration, such as osteoporosis.^3,4^ Interestingly, inflammatory cytokines also prime MSCs to function when MSCs are infused for immunomodulation.^5,6^ In this regard, despite evidence suggesting that MSCs secrete inflammatory cytokines in a proinflammatory functional state,^7^ it remains unclear whether MSC-derived inflammatory cytokines influence their own behaviors under physiological conditions. In this study, we reveal for the first time that MSCs produce and release low levels of TNFα, which is indispensable for the functional maintenance of MSCs. The autocrine/paracrine mode of TNFα regulation of MSCs indicates that MSCs create a beneficial physiological niche for their own homeostasis. Studies have also reported that other inflammatory cytokines, such as IFNγ and IL-6, serve as autocrine regulators of MSCs, but the observations were made during the differentiation process or under inflammatory conditions.^10,25^ Here, we identified that genetic depletion of only *TNFα*, but not *IFNγ* or *IL-6*, results in MSC impairment, suggesting that TNFα has a unique function among inflammatory cytokines to safeguard the stemness of MSCs. Accordingly, our mechanistic experiments reveal that TNFα functions through endocytosis to inhibit mTOR signaling, which is independent of TNFR and TNFR downstream pathways. Our findings indicate for the first time a noninflammatory role of TNFα in governing stem cell fate, paving the way for future studies on physiological TNFα-mediated tissue/organ development and homeostatic control.

To our knowledge, the receptor-independent mechanism(s) mediating the effects of inflammatory cytokines have not been revealed previously. Here, inspired by simple observations of the differential influences of TNFα and TNFR deficiency on MSC homeostasis, as well as the decreased levels of TNFα compared to the concentrations originally added to the MSC culture medium, we discovered that physiological TNFα is endocytosed into MSCs without stimulating TNFR downstream signaling. This novel endocytotic mechanism mediating the effects of TNFα would therefore be considered irrelevant to the canonical role of TNFα as an inflammatory cytokine. Accordingly, at low and high levels, TNFα functions through differential mechanisms to modulate different molecular targets, contributing to stem cell regulation under physiological and pathological conditions, as also indicated in previous studies.^4,15^ Endocytosis integrates nutrient internalization, diverse signal transduction pathways and components in the plasma membrane, thus contributing to cellular and organismal function as a fundamental life process.^33^ For stem cells, it has been reported that clathrin-mediated endocytosis is critical for balancing TGF-β and ERK signaling outputs to regulate embryonic stem cell pluripotency and fate choices and that endocytosis is linked to autophagy to restrict intestinal stem cell proliferation.^34,35^ We have further documented that MSCs use endocytosis to take up apoptotic extracellular vesicles for functional maintenance.^24^ Together with the findings of the present study, these works establish endocytosis as a dynamic yet constitutive mechanism that is indispensable for orchestrating stem cell behaviors. Whether there is a noncanonical mechanism underlying TNFα endocytosis, such as a mechanism associated with inward bending of the membrane to form the endocytic vesicle, remains to be elucidated in future studies.

Rapamycin was first isolated in 1972 from the bacterium *Streptomyces hygroscopicus;* it was initially identified as an antifungal agent and an immunosuppressant and was later discovered to possess antitumor properties.^29^ Subsequently, mTOR signaling was uncovered as the molecular target of rapamycin, with multiple studies revealing mTOR as a master regulator of cells that senses nutrients and growth factors and integrates downstream cascades to coordinate cell growth, metabolism, and autophagy.^29^ It has been increasingly reported that mTOR signaling is involved in the development and maintenance of various stem and progenitor cell populations.^29^ In particular, mTOR regulation of MSCs has yielded conflicting results, with distinct roles of mTORC1 and mTORC2 in MSC lineage commitment.^36^ In this regard, we have previously reported that activation of mTORC1 suppresses RUNX2 expression and promotes PPARγ expression in MSCs, underlying the shift in MSC differentiation from osteogenesis to adipogenesis in osteopenia.^30,37,38^ In the present study, we further discovered that mTORC2 activation in the context of TNFα deficiency and mechanical unloading accompanies mTORC1 activation to result in MSC dysfunction, indicating synergistic roles of mTORC2 and mTORC1 in regulating MSC fate. Importantly, we reveal that endocytosed TNFα binds to mTORC2 to suppress signaling, which represents a novel mechanism of mTOR regulation independent of PI3K, the currently recognized upstream signal of mTOR.^29^ It would be interesting to investigate in future studies whether TNFα control of mTOR equilibrium affects MSC behavioral adaptation to other environmental cues, such as metabolic and autophagic changes in responses to nutrient signals,^29^ thereby coordinating MSC function to maintain tissue/organ homeostasis.

The mechanical properties of the stem cell niche critically influence cell shape and fate decisions, which are mediated by the cytoskeleton and the related mechanosensing pathway.^39^ Changes in the cytoskeleton also contribute to the endocytotic process, which requires dynamic adaptations of the plasma membrane and the transportation of endosomal vesicles.^33^ Not surprisingly, endocytosis has been reported to be influenced by applied forces,^31,32^ which was confirmed in this study for the uptake of TNFα. However, further work is needed to elucidate how mechanical force promotes the endocytosis process and to specify the underlying molecular machinery that responds to force stimuli. Accordingly, it is speculated that loss of mechanical stimuli causes endocytosis failure and MSC dysfunction, indicating that force-driven membrane dynamics are a basic characteristic of living cells. At the organismal level, severe symptoms of skeletal muscle atrophy and bone loss have been observed when individuals are exposed to microgravity during space missions.^19,40^ However, approaches to counteracting mechanical unloading-induced tissue alterations are currently limited. In this study, we used rapamycin, which has been widely applied in the clinic, to effectively protect MSCs and bone homeostasis despite mechanical unloading, highlighting a pharmaceutical therapeutic with osteoanabolic benefits in vivo. Notably, although rapamycin may exert immunosuppressive effects when infused systemically,^29^ we did not observe detrimental side effects during rapamycin application. Instead, the feasibility and effectiveness of using rapamycin to rescue the function of resident MSCs and treat multiple osteopenias have been established by our group, providing a promising therapeutic strategy.^30,37,38^

In summary, we unraveled a previously unrecognized role and mechanism of physiological TNFα that involves dynamic release and endocytosis to safeguard the functional homeostasis of MSCs, and we revealed downstream mTOR signaling as a therapeutic target for counteracting mechanical unloading-induced MSC dysfunction and osteopenia.

## Materials and methods

### Animals

Female C57BL/6J (WT, No. 000664), B6.129S-*Tnf*^*tm1Gkl*^/J (*TNFα*^*−/−*^, No. 003008), B6.129S-*Tnfrsf1a*^*tm1Imx*^ *Tnfrsf1b*^*tm1Imx*^/J (*TNFR*^*−/−*^, No. 003243), B6.129S7-*Ifng*^*tm1Ts*^/J (*IFNγ*^*−/−*^, No. 002287), B6.129S2-*Il6*^*tm1Kopf*^/J (*IL-6*^−^^*/−*^, No. 002650), B6.Cg-Tg(Prrx1-cre)1Cjt/J (*Prx1-Cre*, No. 005584), and B6.Cg-*Gt(ROSA)26Sor*^*tm9(CAG-tdTomato)Hze*^/J (*tdTomato*, No. 007909) mice were purchased from Jackson Laboratory and maintained on a C57BL/6J background for at least ten backcrosses. *Prx1-Cre* and *tdTomato* transgenics were interbred to obtain *Prx1-Cre*;*tdTomato* mice for tracking mesenchymal cells in vivo. Genotyping was performed by PCR using tail samples from mice and primer sequences provided by Jackson Laboratory. Age-matched female littermates were used in all experiments. Female immunocompromised nude mice (Beige *nu/nu* XIDIII) were purchased from Harlan. Mice were housed under pathogen-free conditions, maintained on a standard 12-h light-dark cycle, and given food and water ad libitum. All animal experiments were performed under institutionally approved protocols for animal research (University of Pennsylvania, Protocol No. 805478).

### Hindlimb unloading

WT and *Prx1-Cre*;*tdTomato* mice at 8 weeks of age were subjected to continuous tail suspension for 2 weeks, which was performed according to previous studies.^20^ Briefly, the mice were individually caged and suspended by the tail using a strip of adhesive surgical tape attached to a chain hanging from a pulley. The mice were suspended at a 30° angle to the floor with only the forelimbs touching the floor, allowing the mice to move and access food and water freely. At sacrifice, the hindlimb bones were sampled for the indicated analyses.

### Isolation and culture of mouse MSCs

Isolation and culture of MSCs from mouse bone marrow were performed according to our previous protocol.^22,24^ Briefly, whole bone marrow cells from femora and tibia were seeded, incubated overnight, and rinsed with phosphate-buffered saline (PBS) to remove the nonadherent cells. The adherent cells were cultured with alpha-minimum essential medium supplemented with 20% fetal bovine serum (FBS), 2 mmol·L^−1^ L-glutamine, 55 μmol·L^−^^1^ 2-mercaptoethanol, 100 U·mL^−1^ penicillin, and 100 μg·mL^−1^ streptomycin (all from Invitrogen, USA) at 37 °C in a humidified atmosphere of 5% CO_2_. MSCs were digested with 0.25% trypsin (Invitrogen, USA) and passaged for functional experiments after seeding at appropriate densities.

### Isolation and culture of mouse T cells and macrophages

For culture of mouse T cells, splenocytes were collected and treated with ACK lysis buffer (Lonza, Switzerland) to remove red blood cells. Naïve T cells were isolated with a plate-bound anti-mouse CD3 antibody at 5 μg·mL^−1^ (eBioscience, USA). Activated T cells were obtained by stimulating naïve T cells for 48 h with a soluble anti-mouse CD28 antibody at 2 μg·mL^−1^ (eBioscience, USA) in Dulbecco’s modified Eagle’s medium (DMEM) supplemented with 10% heat-inactivated FBS, 2 mmol·L^−1^ L-glutamine, 55 μmol·L^−1^ 2-mercaptoethanol, 10 mmol·L^−1^ hydroxyethyl piperazine ethanesulfonic acid (HEPES), 1 mmol·L^−1^ sodium pyruvate, 100 U·mL^−1^ penicillin, and 100 μg·mL^−1^ streptomycin (all from Invitrogen, USA). Th1 cells were obtained by treating activated T cells with 20 ng·mL^−1^ IL-2 (PeproTech, USA), 20 ng·mL^−1^ IL-12 (PeproTech, USA), and 10 μg·mL^−1^ anti-IL-4 blocking antibody (BioLegend, USA).

Mouse macrophages were isolated by seeding 2 × 10^6^ femoral ANCs in 6-well plates with 20 ng·mL^−1^ macrophage-colony stimulating factor (M-CSF; PeproTech, USA) in DMEM supplemented with 15% heat-inactivated FBS, 100 U·mL^−1^ penicillin and 100 μg·mL^−1^ streptomycin (all from Invitrogen, USA). After 48 h, nonadherent cells were removed, and the adherent macrophages were cultured for another 48 h with 20 ng·mL^−1^ M-CSF in the presence or absence of 1 ng·mL^−1^ LPS (Millipore, USA).

### Chemical treatments

Chemical reagents and treatments were as follows: recombinant mouse TNFα (PeproTech, USA), mouse RANKL (PeproTech, USA), human TNFα (R&D Systems, USA), and human pro-TNFα (R&D Systems, USA) were added at the indicated concentrations, with a physiological concentration of 1 ng·mL^−1^ used in most experiments. TAPI-2 (Millipore, USA) was used at 120 nmol·L^−1^. Pitstop^®^ 2 (Abcam, UK) was added at 12 μmol·L^−1^. Rapamycin (Abcam, UK) was intraperitoneally administered at 50 nmol·L^−1^ in vitro and 1.5 mg·kg^−1^ per day in vivo for 2 weeks.^30^

### TNFα overexpression and knockdown in vitro

For knockdown of TNFα in vitro, serum-starved MSCs were treated with TNFα siRNA or vehicle siRNA control (Santa Cruz Biotechnology, USA) transfected with the Lipofectamine RNAiMAX reagent (Invitrogen, USA) according to the manufacturers’ instructions. For overexpression of TNFα, green fluorescent protein (GFP)-TNFα fusion protein expression plasmids were kindly provided by Dr. Jennifer Stow (Addgene plasmid No. 28089). Empty plasmids with the same backbone were used as the control. Plasmids were transduced using Lipofectamine LTX with Plus reagent (Life Technologies, USA) according to the manufacturer’s instructions.

### In vitro cyclic stretching

MSC monolayers grown on collagen-coated Flexcell plates were subjected to cyclic stretching (15% elongation at 0.5 Hz) using a Flexcell Fx-4000T tension unit (Flexcell International, USA) for 2 h periods. Control wells were plugged at the bottom by rubber capping without application of any stretches.

### Colony-forming unit (CFU) assay

The formation of fibroblastic colonies (CFU-F) by MSCs was evaluated according to a previous study.^22^ Briefly, a total of 1.5 × 10^6^ all nuclear cells (ANCs) from the bone marrow were seeded in 60 mm culture dishes and cultured for 16 days. The colonies were washed with PBS, fixed with 2% paraformaldehyde (PFA; Sigma-Aldrich, USA), and stained with 0.5% toluidine blue solution (Sigma-Aldrich, USA). The number of cell colonies was counted under a microscope, and groups of more than 50 cells were considered colonies.

### BrdU cell proliferation assay

Analysis of MSC proliferation was performed using BrdU labeling.^24^ MSCs were seeded onto 8-well chamber slides (Thermo Fisher Scientific, USA) at a concentration of 2 × 10^4^ cells per well. After adherence, BrdU labeling reagent (Invitrogen, USA) was added to the medium at 1:100 for 48 h. The cells were then fixed with 70% ethanol, denatured with 2 N HCl, and stained with a BrdU Staining Kit (Invitrogen, USA) according to the manufacturer’s instructions. Fluoroshield mounting medium with DAPI (Abcam, UK) was used for counterstaining and mounting. The BrdU-positive cells in five fields of view from each sample were quantified using ImageJ software (National Institute of Health, USA) and are presented relative to the total number of cells.

### Osteogenic differentiation

For analysis of osteogenic differentiation capability,^22^ MSCs were cultured in osteogenic inductive medium including 2 mmol·L^−1^ β-glycerophosphate (Sigma-Aldrich, USA), 100 μmol·L^−1^ L-ascorbic acid phosphate (Wako, Japan), and 10 nmol·L^−1^ dexamethasone (Sigma-Aldrich, USA). After 4 weeks of induction, in vitro mineralization was detected by 1% Alizarin Red S (Sigma-Aldrich, USA) staining, and the positively stained areas were quantified as percentages of the total area using ImageJ software (National Institute of Health, USA). Protein expression levels of osteogenic marker genes were also examined by a Western blot assay, as described below.

### Adipogenic differentiation

For adipogenic differentiation,^24^ MSCs were cultured in adipogenic inductive medium containing 500 nmol·L^−1^ isobutylmethylxanthine (Sigma-Aldrich, USA), 60 μmol·L^−^^1^ indomethacin (Sigma-Aldrich, USA), 500 nmol·L^−^^1^ hydrocortisone (Sigma-Aldrich, USA), 10 μg·mL^−^^1^ insulin (Sigma-Aldrich, USA), and 100 nmol·L^−1^ L-ascorbic acid phosphate (Wako, Japan). After 7 days of induction, lipid droplets were stained with Oil Red O (Sigma-Aldrich, USA), and the positively stained cells were quantified as percentages of the total cells using ImageJ software (National Institute of Health, USA). Protein expression levels of adipogenic marker genes were also examined by a Western blot assay, as described below.

### Ectopic tissue formation

Ectopic tissue formation was performed to analyze MSC function in vivo, as previously stated.^24^ MSCs (4 × 10^6^) were mixed with 40 mg of hydroxyapatite/tricalcium phosphate ceramic powder (Zimmer Inc., USA) as a carrier and were subcutaneously implanted into 8-week-old immunocompromised mice. At 8 weeks after implantation, the implants were harvested, fixed in 4% PFA, decalcified with 10% ethylenediaminetetraacetic acid (EDTA, pH 7.4), embedded in paraffin, and sectioned into 6 μm slices. The sections were then stained with hematoxylin and eosin, and de novo formed bone areas were analyzed using ImageJ software (National Institute of Health, USA) and shown as percentages of the total area.

### Endocytosis analysis

Recombinant mouse TNFα (PeproTech, USA), human TNFα (R&D Systems, USA), and human pro-TNFα (R&D Systems, USA) were labeled with FITC using the ProtOn^TM^ Fluorescein Labeling Kit (Vector Laboratories, Italy) according to the manufacturer’s instructions. FITC-labeled TNFα and pro-TNFα were then used to treat MSCs on coverslips at 1 ng·mL^−1^ for 24 h. The cells were then fixed in 4% PFA, and the coverslips were mounted using Fluoroshield Mounting Medium with DAPI (Abcam, UK). The FITC-positive cells from five fields per group were quantified and are shown as percentages of the total cell number. FITC intake rates were analyzed using flow cytometric analysis, as described below.

### Flow cytometric analysis

For analysis of in situ MSCs, ANCs from the hindlimbs of *Prx1-Cre*;*tdTomato* mice were collected and stained with a FITC anti-mouse TNFα antibody (BioLegend, USA) at 1:100 or a PerCP anti-mouse p-mTOR antibody (Thermo Fisher Scientific, USA) at 1:100 for 60 min on ice using Intracellular Staining Permeabilization Wash Buffer (BioLegend, USA). For analysis of MSC endocytosis, MSCs were collected after FITC-labeled TNFα and pro-TNFα treatments and fixed in 2% PFA. For analysis of MSC surface markers, cultured MSCs were collected and stained with PE-conjugated antibodies against CD73 (Thermo Fisher Scientific, USA), CD90 (BioLegend, USA), CD105 (Thermo Fisher Scientific, USA), CD146 (Thermo Fisher Scientific, USA), CD166 (Thermo Fisher Scientific, USA), Stem cell antigen 1 (Sca1; BD Biosciences, USA), and CD45 (Thermo Fisher Scientific, USA) and a PerCP-conjugated antibody against CD34 (BioLegend, USA) at 1:100 for 60 min on ice. All samples were analyzed using FACS^Calibur^ with CellQuest software (BD Bioscience, USA).

### qRT-PCR

Total RNA was isolated from cultured cells using the miRNeasy Mini Kit (Qiagen, Germany) according to the manufacturer’s instructions. cDNA was synthesized using SuperScript III (Life Technologies, USA). Real-time PCR was performed using SYBR Green Supermix (Bio-Rad, USA) on a CFX96^TM^ Real-Time PCR System (Bio-Rad, USA). The primers for mouse *TNFα* were as follows: forward, 5′-CCTGTAGCCCACGTCGTAG-3′; reverse, 5′-GGGAGTAGACAAGGTACAACCC-3′. The primers for mouse *GAPDH* were as follows: forward, 5′-TGTGTCCGTCGTGGATCTGA-3′; reverse, 5′-TTGCTGTTGAAGTCGCAGGAG-3′.

### Western blot

Western blot assays were performed according to previous studies.^22,24^ Cultured MSCs were lysed in RIPA Lysis Buffer with protease and phosphatase inhibitors (Santa Cruz Biotechnology, USA). Protein levels were quantified using the Pierce^TM^ BCA Protein Assay Kit (Thermo Fisher Scientific, USA). A total of 20 μg of protein was separated by SDS-PAGE (Invitrogen, USA) and transferred to 0.2 μm nitrocellulose membranes (Millipore, USA). The membranes were then blocked with 5% nonfat dry milk and 0.1% Tween-20 for 1 h, followed by incubation overnight at 4 °C with the following primary antibodies: antibodies against cJun, p-p53, p53, p-NFκB (p50), NFκB (p50), osteocalcin (OCN), poly (ADP-ribose) polymerase (PARP), and peroxisome proliferator-activated receptor gamma (PPARγ) were purchased from Santa Cruz Biotechnology, USA, and were used at concentrations of 1:200; antibodies against TNFR2, runt-related transcription factor 2 (RUNX2), PI3K, PTEN, p-mTOR, mTOR, p-Akt, Akt, Caspase 3, p-cJun, cJun N-terminal kinase (JNK), p-JNK, p-NFκB (p65), NFκB (p65), p-p38, p38, active-β-catenin, β-catenin, p-ERK1/2, ERK1/2, p-P70S6K, P70S6K, p-Rictor, p-Raptor, Raptor and p-Sin1 were obtained from Cell Signaling Technology, USA, and were used at concentrations of 1:1 000; the antibody against lipoprotein lipase was purchased from Thermo Fisher Scientific, USA, and was used at a concentration of 1:1 000; antibodies against TNFR1, alkaline phosphatase, p-Smad3, Smad3, and GFP were purchased from Abcam, UK, and were used at concentrations of 1:1 000; antibodies against Rictor and Sin1 were purchased from Bethyl Laboratories, USA, and were used at concentrations of 1:1 000; and the antibody against β-Actin was purchased from Sigma-Aldrich, USA, and was used at a concentration of 1:1 000. The membranes were then washed and incubated at room temperature for 1 h with horseradish peroxidase-conjugated secondary antibodies (Santa Cruz Biotechnology, USA). Immunoreactive proteins were detected using SuperSignal^TM^ West Pico PLUS Chemiluminescent Substrate, SuperSignal^TM^ West Femto Maximum Sensitivity Substrate (Thermo Fisher Scientific, USA) and Autoradiography Film (Labscientific Inc., USA). All Western blot data were verified by more than three independent experiments.

### ELISA

Supernatants of cell culture media and bone marrow were collected after centrifugation at 12 000 r·min^−1^ for 15 min. Concentrations of TNFα were analyzed using a Mouse ELISA MAX^TM^ Deluxe kit (BioLegend, USA) according to the manufacturer’s instructions.

### Co-IP assay

Cultured MSCs were lysed in RIPA Lysis Buffer with protease and phosphatase inhibitors (Santa Cruz Biotechnology, USA). Protein levels were quantified using the Pierce^TM^ BCA Protein Assay Kit (Thermo Fisher Scientific, USA). One microgram of the control IgG, together with 20 μL of resuspended protein A/G PLUS-agarose, was added to protein lysates and incubated at 4 °C for 30 min. The beads were pelleted by centrifugation at 2 500 r·min^−1^ for 5 min at 4 °C, and the supernatants containing total cellular protein (450 μg) were collected. Primary antibodies were added at 1:100 and incubated overnight at 4 °C followed by incubation with 20 μL of resuspended protein A/G PLUS-agarose for 2 h. Immunoprecipitates were then collected after centrifuging at 2 500 r·min^−1^ for 5 min at 4 °C and were resuspended in 30 μL of electrophoresis sample buffer. Ten-microliter aliquots were subjected to Western blot analysis. All Co-IP data were verified by more than three independent experiments.

### mTORC1 activity analysis

Whole-cell lysates were collected from MSCs followed by co-IP with an anti-mTOR antibody. Purified and enriched mTOR complexes were used to measure kinase activity with the K-LISA^TM^ mTOR Activity Kit (Millipore, USA) according to the manufacturer’s instructions.

### Calcein labeling assay

Calcein labeling histomorphometric analysis was performed as reported previously.^22^ Mice were intraperitoneally injected with calcein (Sigma-Aldrich, USA) at 15 mg·kg^−1^ prepared in 2% sodium bicarbonate solution at 10 days and 3 days before sacrifice. Bone formation analyses using MAR and BFR were performed according to the standardized nomenclature for bone histomorphometry under a fluorescence microscope (IX71; Olympus, Japan).

### Bone marrow adiposity analysis

Femora were fixed in 4% PFA and decalcified with 10% EDTA (pH 7.4), followed by cryosectioning and staining with Oil Red O solution. Bone marrow adipocytes surrounding the trabecular areas were analyzed. Positively stained areas were quantified using ImageJ software (National Institute of Health, USA) and are shown as percentages of the total area.

### Micro-CT analysis

At sacrifice, the femora were fixed in 4% PFA and scanned using a high-resolution Scanco μCT35 scanner (Scanco Medical AG, Switzerland) with a voxel size of 20 μm at 70 kV and 200 μA. Images were then reconstructed, and data were analyzed by measuring BMD and BV/TV.

### Statistics

All data are represented as the mean ± standard deviation. Comparisons between two groups were analyzed using independent unpaired two-tailed Student’s *t* tests, and comparisons among multiple groups were analyzed using one-way analysis of variance with Bonferroni post hoc tests. *P* values < 0.05 were considered statistically significant.

## Supplementary information

Supplementary material

## References

[1] Uccelli A, Moretta L, Pistoia V (2008). Mesenchymal stem cells in health and disease. Nat. Rev. Immunol..

[2] Wang Y, Chen X, Cao W, Shi Y (2014). Plasticity of mesenchymal stem cells in immunomodulation: pathological and therapeutic implications. Nat. Immunol..

[3] Liu Y (2011). Mesenchymal stem cell-based tissue regeneration is governed by recipient T lymphocytes via IFN-gamma and TNF-alpha. Nat. Med..

[4] Wang L (2013). IFN-gamma and TNF-alpha synergistically induce mesenchymal stem cell impairment and tumorigenesis via NFkappaB signaling. Stem Cells.

[5] Ren G (2008). Mesenchymal stem cell-mediated immunosuppression occurs via concerted action of chemokines and nitric oxide. Cell Stem Cell.

[6] Xu G, Zhang Y, Zhang L, Roberts AI, Shi Y (2009). C/EBPbeta mediates synergistic upregulation of gene expression by interferon-gamma and tumor necrosis factor-alpha in bone marrow-derived mesenchymal stem cells. Stem Cells.

[7] Waterman RS, Tomchuck SL, Henkle SL, Betancourt AM (2010). A new mesenchymal stem cell (MSC) paradigm: polarization into a pro-inflammatory MSC1 or an Immunosuppressive MSC2 phenotype. PLoS ONE.

[8] Haynesworth SE, Baber MA, Caplan AI (1996). Cytokine expression by human marrow-derived mesenchymal progenitor cells in vitro: effects of dexamethasone and IL-1 alpha. J. Cell Physiol..

[9] Kinnaird T (2004). Marrow-derived stromal cells express genes encoding a broad spectrum of arteriogenic cytokines and promote in vitro and in vivo arteriogenesis through paracrine mechanisms. Circ. Res..

[10] Ke F (2014). Autocrine interleukin-6 drives skin-derived mesenchymal stem cell trafficking via regulating voltage-gated Ca(2+) channels. Stem Cells.

[11] Aggarwal BB, Gupta SC, Kim JH (2012). Historical perspectives on tumor necrosis factor and its superfamily: 25 years later, a golden journey. Blood.

[12] Akash MSH, Rehman K, Liaqat A (2018). Tumor necrosis factor-alpha: role in development of insulin resistance and pathogenesis of type 2 diabetes mellitus. J. Cell Biochem..

[13] Black RA (1997). A metalloproteinase disintegrin that releases tumour-necrosis factor-alpha from cells. Nature.

[14] Hohmann HP, Remy R, Poschl B, van Loon AP (1990). Tumor necrosis factors-alpha and -beta bind to the same two types of tumor necrosis factor receptors and maximally activate the transcription factor NF-kappa B at low receptor occupancy and within minutes after receptor binding. J. Biol. Chem..

[15] Huang H (2011). Dose-specific effects of tumor necrosis factor alpha on osteogenic differentiation of mesenchymal stem cells. Cell Prolif..

[16] Sui BD, Hu CH, Zheng CX, Jin Y (2016). Microenvironmental views on mesenchymal stem cell differentiation in aging. J. Dent. Res..

[17] Ozcivici E (2010). Mechanical signals as anabolic agents in bone. Nat. Rev. Rheumatol..

[18] Vining KH, Mooney DJ (2017). Mechanical forces direct stem cell behaviour in development and regeneration. Nat. Rev. Mol. Cell Biol..

[19] Klein-Nulend J, Bacabac RG, Veldhuijzen JP, Van Loon JJ (2003). Microgravity and bone cell mechanosensitivity. Adv. Space Res.

[20] Wang J (2016). Mechanical stimulation orchestrates the osteogenic differentiation of human bone marrow stromal cells by regulating HDAC1. Cell Death Dis..

[21] Li P (2015). Simulated microgravity disrupts intestinal homeostasis and increases colitis susceptibility. FASEB J..

[22] Yang R (2018). Tet1 and Tet2 maintain mesenchymal stem cell homeostasis via demethylation of the P2rX7 promoter. Nat. Commun..

[23] Sui B (2016). Mesenchymal progenitors in osteopenias of diverse pathologies: differential characteristics in the common shift from osteoblastogenesis to adipogenesis. Sci. Rep..

[24] Liu D (2018). Circulating apoptotic bodies maintain mesenchymal stem cell homeostasis and ameliorate osteopenia via transferring multiple cellular factors. Cell Res..

[25] Duque G (2009). Autocrine regulation of interferon gamma in mesenchymal stem cells plays a role in early osteoblastogenesis. Stem Cells.

[26] Wada T, Nakashima T, Hiroshi N, Penninger JM (2006). RANKL-RANK signaling in osteoclastogenesis and bone disease. Trends Mol. Med..

[27] Kenny PA, Bissell MJ (2007). Targeting TACE-dependent EGFR ligand shedding in breast cancer. J. Clin. Invest..

[28] Nandadasa S (2019). Secreted metalloproteases ADAMTS9 and ADAMTS20 have a non-canonical role in ciliary vesicle growth during ciliogenesis. Nat. Commun..

[29] Meng D, Frank AR, Jewell JL (2018). mTOR signaling in stem and progenitor cells. Development.

[30] Chen C (2015). mTOR inhibition rescues osteopenia in mice with systemic sclerosis. J. Exp. Med..

[31] Raucher D, Sheetz MP (1999). Membrane expansion increases endocytosis rate during mitosis. J. Cell Biol..

[32] Kiyoshima D, Kawakami K, Hayakawa K, Tatsumi H, Sokabe M (2011). Force- and Ca(2)(+)-dependent internalization of integrins in cultured endothelial cells. J. Cell Sci..

[33] Kumari S, Mg S, Mayor S (2010). Endocytosis unplugged: multiple ways to enter the cell. Cell Res..

[34] Narayana YV, Gadgil C, Mote RD, Rajan R, Subramanyam D (2019). Clathrin-mediated endocytosis regulates a balance between opposing signals to maintain the pluripotent state of embryonic stem cells. Stem Cell Rep..

[35] Zhang P (2019). An SH3PX1-dependent endocytosis-autophagy network restrains intestinal stem cell proliferation by counteracting EGFR-ERK signaling. Dev. Cell.

[36] Martin SK (2015). Brief report: the differential roles of mTORC1 and mTORC2 in mesenchymal stem cell differentiation. Stem Cells.

[37] Chen C (2017). Mesenchymal stem cell transplantation in tight-skin mice identifies miR-151-5p as a therapeutic target for systemic sclerosis. Cell Res..

[38] Liu Y (2016). Chronic high dose alcohol induces osteopenia via activation of mTOR signaling in bone marrow mesenchymal stem cells. Stem Cells.

[39] Halder G, Dupont S, Piccolo S (2012). Transduction of mechanical and cytoskeletal cues by YAP and TAZ. Nat. Rev. Mol. Cell Biol..

[40] Steffen JM, Musacchia XJ (1986). Spaceflight effects on adult rat muscle protein, nucleic acids, and amino acids. Am. J. Physiol..

