# Supplementary material for: Mechanical force-driven TNFα endocytosis governs stem cell homeostasis
Source: Bone Res. 2021 Jan 1;8:44. doi: 10.1038/s41413-020-00117-x (PMC7775432; doi:10.1038/s41413-020-00117-x)
Supplement: Supplementary file 1 — Supplementary material [file 41413_2020_117_MOESM1_ESM.pdf]

Supplemental Figures and Figure Legends

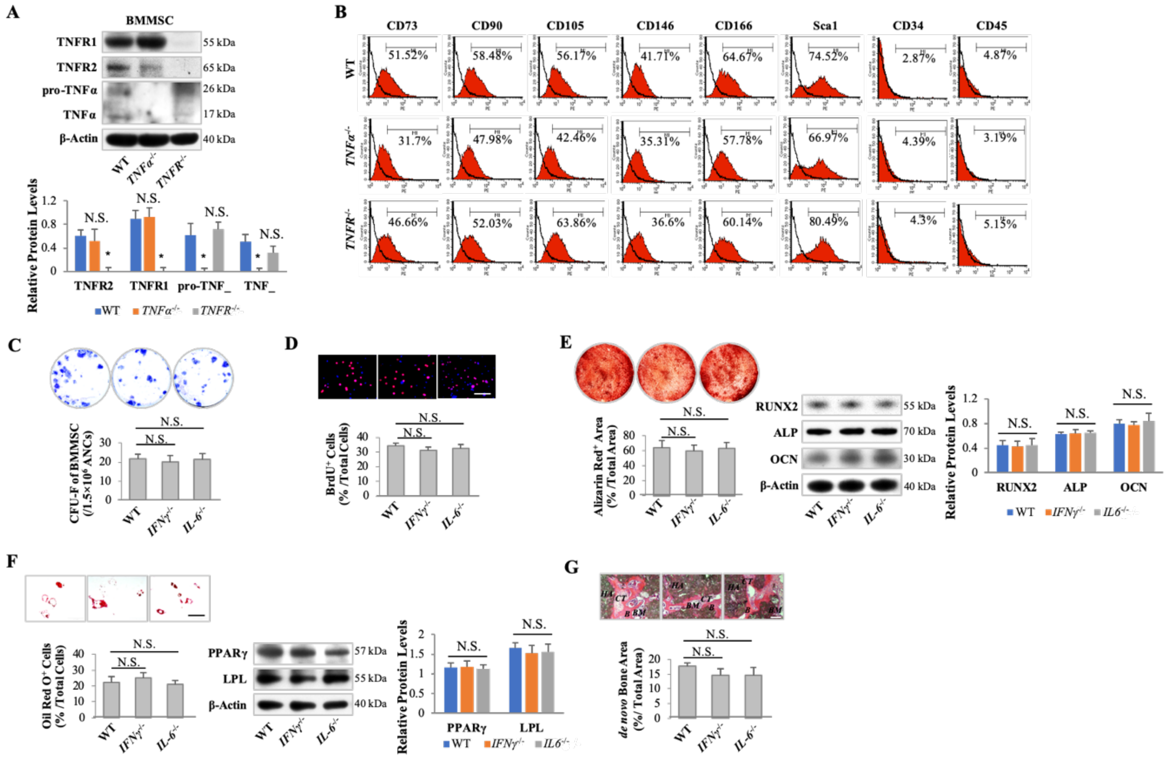

Fig. S1. Characterization of MSCs derived from the genetic mouse models. (**A**) Western blot analyses of *TNFα* and *TNFR* gene knockout efficiency (*N* = 3). (**B**) Flow cytometric analyses of surface markers of MSCs derived from WT, $TNF\alpha^{-/-}$ and $TNFR^{-/-}$ mice (*N* = 3). (**C-G**) Functional analyses of MSCs according to CFU, BrdU labeling, osteogenic and adipogenic differentiation, and ectopic tissue formation. MSCs were derived from WT, $IFN\gamma^{-/-}$ or $IL\text{-}6^{-/-}$ mice (*N* = 3). B, bone; CT, connective tissue; BM, bone marrow; HA, implanted scaffolds. Scale bars = 100 μm. For quantification of Western blotting, two-tailed Student t test was used for the comparison between treatment and WT group. N.S., not significant. Data represent mean ± SD.

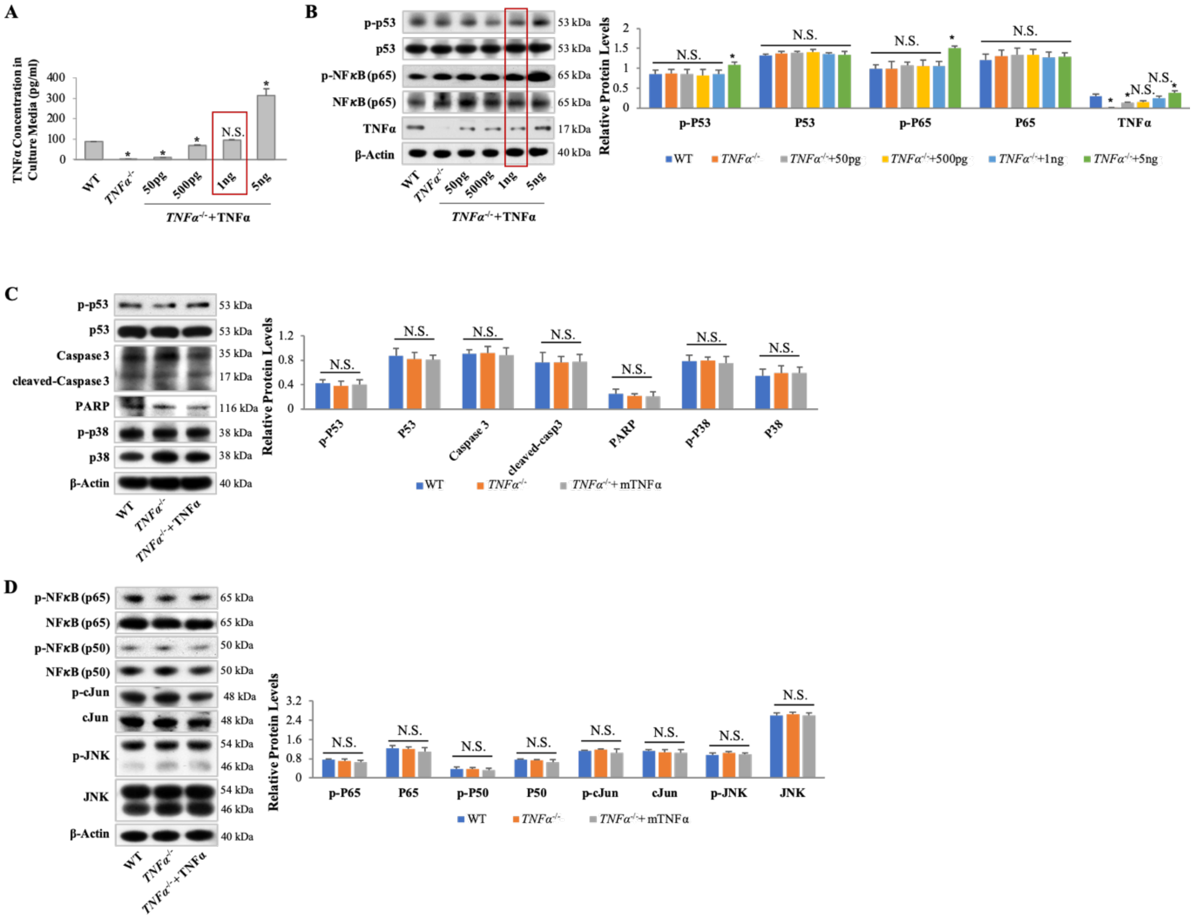

Fig. S2. Physiological level of TNFα does not induce receptor-mediated signaling responses. (**A**) ELISA analysis of secreted TNFα levels by MSCs. *N* = 3. TNFα was added at different doses. (**B**) Western blot analyses of TNFR downstream signaling. *N* = 3. TNFα was added at different doses. (**C, D**) Western blot analyses of TNFR downstream signaling. *N* = 3. TNFα was added at 1 ng/ml. For quantification of Western blotting, two-tailed Student t test was used for the comparison between treatment and WT group. *, $P < 0.05$. N.S., not significant. Data represent mean ± SD.

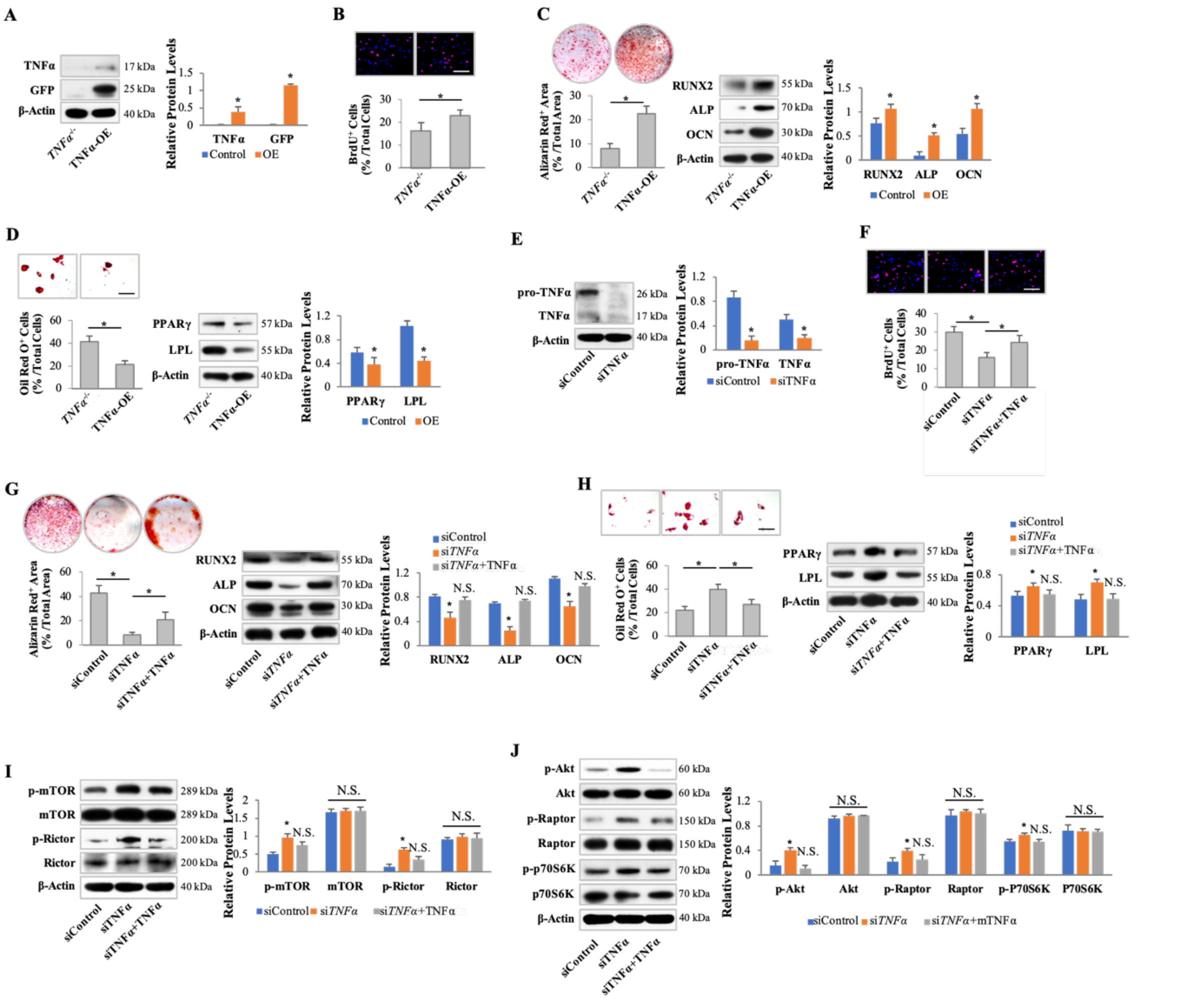

Fig. S3. TNFα regulates MSC homeostasis *in vitro*. (**A**) Western blot analysis of TNFα overexpression (OE) efficiency in *TNFα*$^{-/-}$ MSCs (*N* = 3). (**B-D**) Functional analyses of MSCs according to BrdU labeling, osteogenic and adipogenic differentiation (*N* = 3). (**E**) Western blot analysis of TNFα knockdown efficiency in WT MSCs (*N* = 3). (**F-H**) Functional analyses of MSCs according to BrdU labeling, osteogenic and adipogenic differentiation. *N* = 3. TNFα was added at 1 ng/ml. (**I, J**) Western blot analyses of mTOR signaling (*N* = 3). For quantification of Western blotting, two-tailed Student t test was used for the comparison between treatment and control group. *, $P < 0.05$. N.S., not significant. Data represent mean ± SD.

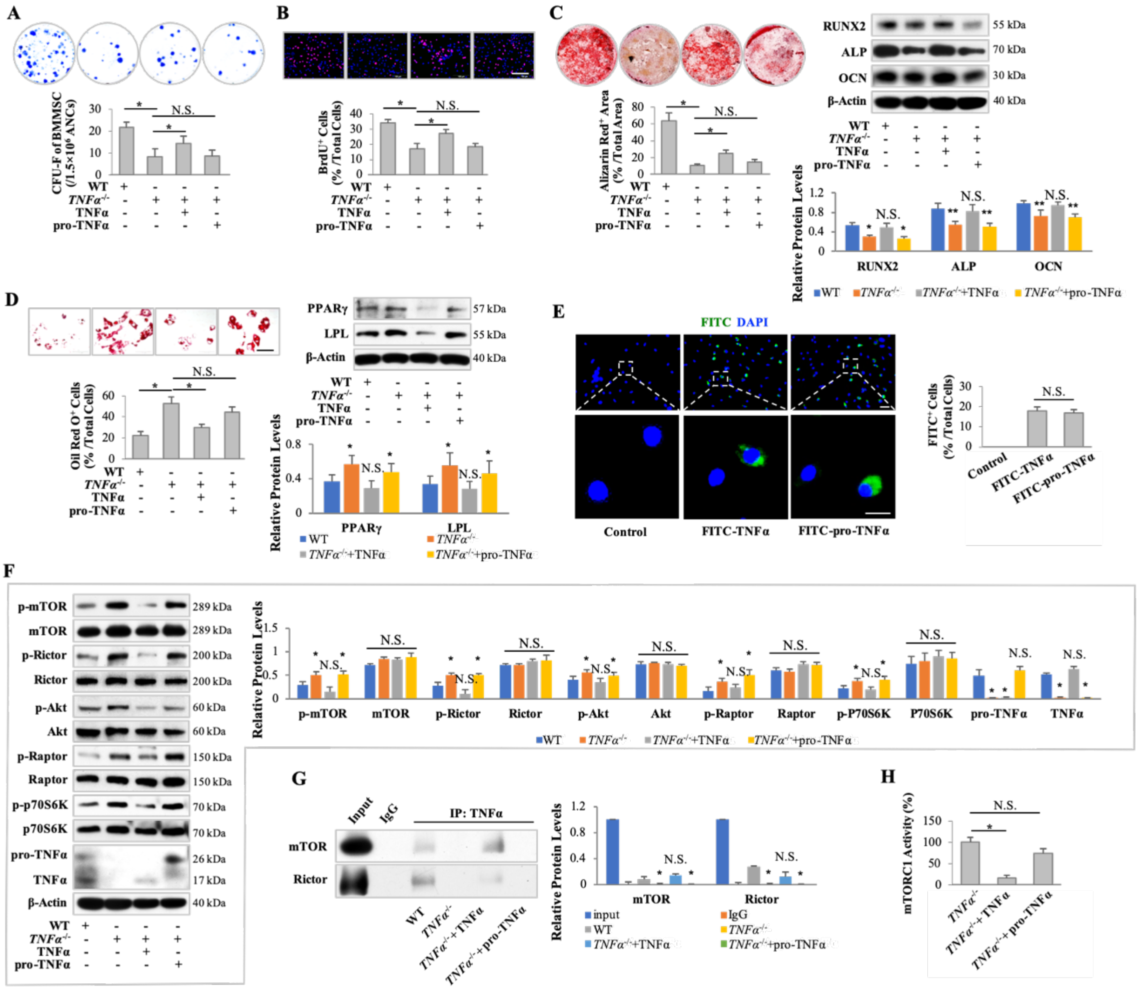

Fig. S4. Pro-TNFα fails to regulate MSC function despite endocytosis. (**A-D**) Functional analyses of MSCs according to CFU, BrdU labeling, osteogenic and adipogenic differentiation. *N* = 3. Scale bars = 100 μm. (**E**) Endocytosis analysis of FITC-labeled TNFα or pro-TNFα uptaken by MSCs for 24 h *in vitro*. *N* = 3. Scale bars = 20 μm (up) and 7 μm (bottom). (**F**) Western blot analyses of mTOR signaling (*N* = 3). (**G**) Co-IP analysis of binding of TNFα and pro-TNFα to mTOR complex components (*N* = 3). (**H**) Analysis of mTORC1 activity using an ELISA-based assay (*N* = 3). TNFα and pro-TNFα were added at 1 ng/ml, respectively. For quantification of Western blotting, two-tailed Student t test was used for the comparison between treatment and WT group. *, P < 0.05. N.S., not significant. Data represent mean ± SD.

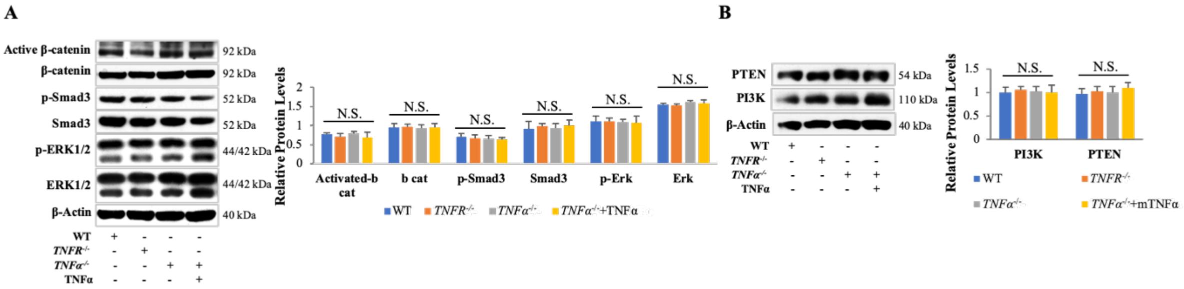

Fig. S5. TNFα exerts limited effects on canonical Wnt, TGF-β or ERK pathways, nor the PI3K pathway upstream of Akt. (**A, B**) Western blot analyses of multiple signaling pathways in MSCs. $N = 3$. TNFα was added at 1 ng/ml. For quantification of Western blotting, two-tailed Student t test was used for the comparison between treatment and WT group. *, $P < 0.05$. N.S., not significant. Data represent mean ± SD.

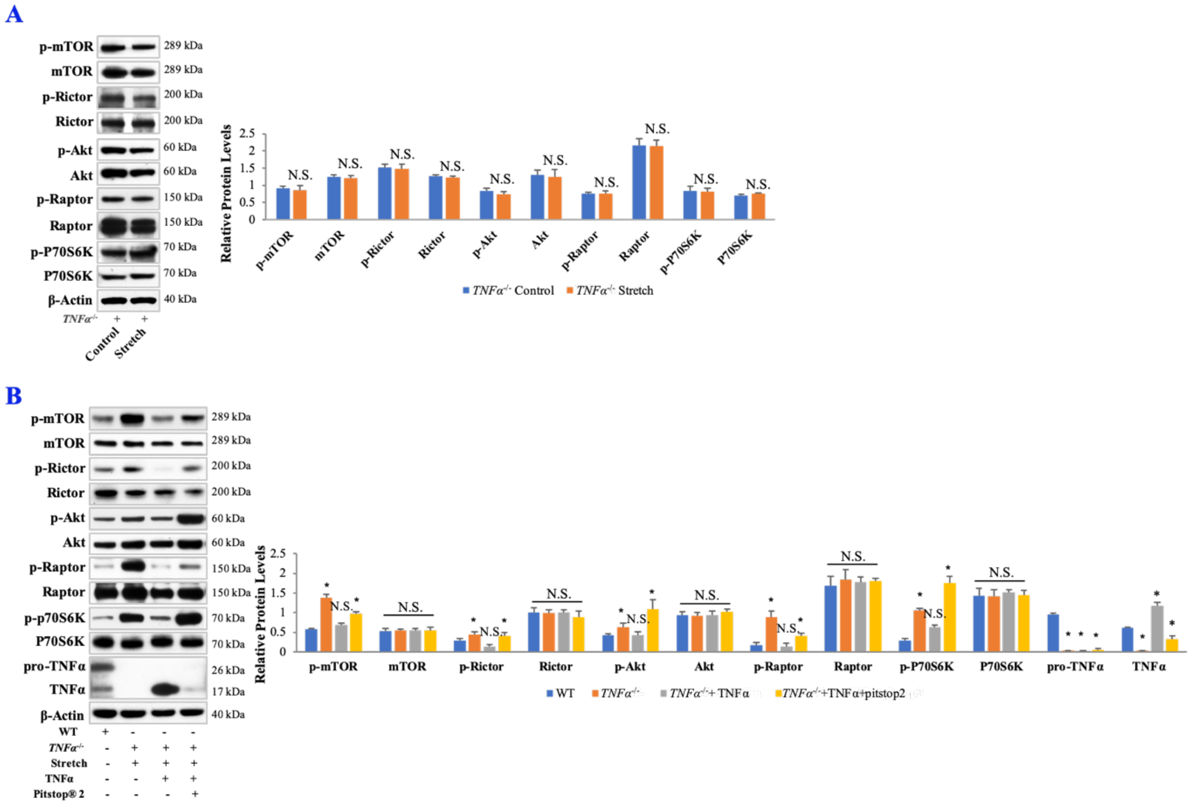

Fig. S6. Mechanical stretching restrains mTOR signaling *via* TNFα endocytosis. (**A, B**) Western blot analysis of mTOR signaling in MSCs. $N = 3$. TNFα and Pitstop® 2 were added at 1 ng/ml and 12 μM, respectively. For quantification of Western blotting, two-tailed Student t test was used for the comparison between treatment and WT/control group. *, $P < 0.05$. N.S., not significant. Data represent mean ± SD.
